# The influence of seasonal climate variability on mortality in pre-industrial Sweden

**DOI:** 10.3402/gha.v6i0.20153

**Published:** 2013-04-03

**Authors:** Barbara Schumann, Sören Edvinsson, Birgitta Evengård, Joacim Rocklöv

**Affiliations:** 1 Department of Public Health and Clinical Medicine, Umeå Centre for Global Health Research, Umeå University, Umeå, Sweden; 2 Ageing and Living Conditions Programme, Centre for Population Studies, Umeå University, Umeå, Sweden; 3 Demographic Database, Umeå University, Umeå, Sweden; 4 Department of Clinical Microbiology, Umeå University, Umeå, Sweden

**Keywords:** climate variability, mortality, Sweden, pre-industrial period, urban population

## Abstract

**Background:**

Recent studies have shown an association between weather and climatic factors with mortality, cardiovascular and infectious diseases. We used historical data to investigate the impact of seasonal temperature and precipitation on total mortality in Uppsala, Sweden, during the first two stages of the demographic transition, 1749–1859.

**Design:**

We retrieved mortality and population numbers of the Uppsala Domkyrka parish from digitised parish records and obtained monthly temperature and precipitation measures recorded in Uppsala during that time. Statistical models were established for year-to-year variability in deaths by annual and seasonal temperature and precipitation, adjusting for longer time trends. In a second step, a model was established for three different periods to study changes in the association of climate variability and mortality over time. Relative risks (RR) with 95% confidence intervals (CI) were calculated.

**Results:**

Precipitation during spring and autumn was significantly associated with annual mortality (spring RR 0.982, CI 0.965–1.000; autumn RR 1.018, CI 1.004–1.032, respectively, per centimetre increase of precipitation). Higher springtime temperature decreased annual mortality, while higher summer temperature increased the death toll; however, both were only borderline significant (*p*=0.07). The significant effect of springtime precipitation for mortality was present only in the first two periods (1749–1785 and 1786–1824). On the contrary, the overall effect of autumn precipitation was mainly due to its relevance during the last period, 1825–1859 (RR 1.024, CI 0.997–1.052). At that time, higher winter precipitation was found to decrease mortality.

**Conclusions:**

In urban Uppsala, during the 18th and 19th century, precipitation appeared to be a stronger predictor for mortality than temperature. Higher spring precipitation decreased and higher autumn precipitation increased the number of deaths. However, this association differed before and during the early stages of industrialisation. Further research shall take age-specific differences into account, as well as changes in socio-economic conditions during that time.

Sweden went through fundamental changes from the 18th and early 19th century to the turn of the 20th century, when the country developed from a predominantly agricultural society to one characterised by rapid industrial development. The turning point occurred around 1850, exemplified by economic reforms facilitating industrial development. The utilisation of new technology was another important factor; in 1849 the first steam-driven sawmill was established in Tunadal outside Sundsvall (1). The number and size of towns increased considerably due to migration of rural landless people to urban areas and general population growth (2). Compared to other European countries, industrialisation came late to Sweden. Still during most of the 19th century, Sweden was a predominantly agricultural country. Around 1800, 90% of the population lived in rural areas; this proportion did not change much before the middle of the 19th century (3). Both fertility and mortality rates were high, especially in young children, and infectious diseases were by far the most frequent cause of death (2). Parallel to the process of urbanisation and industrialisation, Sweden also went through changes in agricultural practices, trading, and occupational patterns. Agricultural productivity was increased, while at the same time, the dependency on regional and national food production decreased (2). These changes led in the late 1800s to an improved standard of living and less dependence on harvest outcomes.

The average life expectancy increased in men from 32.9 years in the mid-18th century to 43.9 years 100 years later; in the beginning of the 20th century, it was 58.8 years. The respective ages for women were 36.2, 47.4, and 62.2 years (4). The main reason was a drastic reduction in infant mortality, owing to improved sanitary conditions and nutritional status (2, 5, 6). In the 19th century, Sweden moved from the first to the second stage of the demographic transition, characterised by decreased mortality, especially in infants and young children (6).

## Social factors and health

Despite the improved standard of living in urban places, labour conditions in the emerging factories were harsh, with long working hours at hazardous, unhealthy work places. Homes in the growing towns and cities were overcrowded and had poor sanitary conditions. Crowding of people and more exchange in goods facilitated the spread of infectious agents. This is the main reason why before and in the early years of industrialisation, mortality was on average higher in urban than in rural areas in Sweden (7, 8). Still, many diseases were related to poverty, poor hygienic conditions, and overcrowding. Even today, social factors such as occupation and income are predictive for a range of chronic and communicable diseases as well as for mortality, mainly through the association of social status with disease risk factors (9, 10).

## The impact of weather and climate on health

Climate and weather factors can have an impact on mortality either directly through physiological reactions to cold or heat, or through intermediate factors such as crop yields (affecting nutritional status) and transmission of infectious agents and diseases (11). Apart from individual biological and lifestyle-related causes as well as societal and economic factors, a number of climatic and environmental variables are related to disease risk and mortality. Most studies to date have been conducted on weather variability in relation to health outcomes, addressing thus short-term (daily and seasonal) variability of meteorological conditions. Less has been researched on the annual (year-to-year) variability that is referred to as climate, or more precisely climate variability. Some researchers have also investigated the health impact of cyclic variations of climate in phenomena such as the El Nino or the North Atlantic Oscillation. Messner (12) showed for northern Sweden a strong association between the Arctic Oscillation and myocardial infarctions with a lag of 3 days. On the contrary, for the zoonotic disease nephropathia epidemica (vole fever), no clear impact of the North Atlantic Oscillation could be found (13).

Based on modern data, a seasonal pattern of mortality has been observed, the highest numbers of deaths occurring normally during the cold season (14). Cold weather contributes to a wide range of public health impacts, including respiratory and cardiovascular events and mortality (15). Rocklov et al. (16, 17) found an association of decreasing ambient temperatures with increasing cardiovascular mortality rates in today's Sweden.

In the last centuries, wealth and health in Sweden were to a large degree dependent on regional or national agricultural production, which was prone to high fluctuations due to weather-related harvest failures, albeit to a decreasing degree towards the end of the 19th century. Poor nutritional conditions made the population more vulnerable to infectious diseases. War and crop failures in the 18th century led to famines and consequently high incidences of infectious diseases. In the 19th century, Sweden suffered from several cholera outbreaks with high number of deaths over a short period (2, 18).

During the last centuries, climatic fluctuations and trends accompanied considerable economic, social, and demographic changes. After the medieval warm period, Eurasia experienced a ‘little ice age’ that started around 1300 and ended in the late 19th century (18–20). Winters were on average about 2–3°C colder than today, and precipitation patterns changed, although there were also large inter-regional differences in climatic trends. According to McMichael (20), during years with temperatures below the long-term mean of the period, there was a 35% higher risk of epidemics in China, and Ljungqvist (18) reported decreasing life expectancies and disease outbreaks in Europe during colder periods after the warmer medieval period.

## Research gaps

Anthropogenic climate change due to industrialisation followed the ‘little ice age’ in the 20th century, leading to increasing average temperature and increased precipitation in some regions, particularly the northern regions (21–23). Today, awareness of global warming has led to increased interest in the role of climatic factors for health.

The extent to which the colder average temperature during the ‘little ice age’ was associated with increased mortality in Sweden is not known. It is also necessary to disentangle the effect of climate change over several centuries from socio-economic changes occurring at the same time.

Most research so far focuses on the relationship between short-term changes in weather (weather variability) and human health. A range of studies using modern data have been conducted on the role of ambient temperature and precipitation for human health and mortality both in high- and low-income countries (11, 16, 17). Few studies deal with cyclic climate patterns such as the North Atlantic Oscillation (12, 13), and, to our knowledge, none of them investigated their health impact in pre-industrial populations. There are also long-term trends in climate (climate change occurring over years, decades, centuries, or millennia) of either natural or human origin. Due to limited availability of reliable health data over longer time periods, there is a need for research on health impacts of climate variability and climate change directly. To our knowledge, no empirical studies have been performed about the climate–mortality association in pre-industrial Europe.

## Research question

The aim of this study was to analyse the association between climatic variability and annual mortality in urban Uppsala, Sweden, between 1749 and 1859 (i.e. before and during the early stages of industrialisation). We were in particular interested in the association between seasonal temperature and precipitation with annual mortality.

## Methods

### Population data

We used parish registers (‘Tabellverket’) of the Demographic Database (www.ddb.umu.se) at Umeå University, containing annual population data aggregated at the parish level. The establishment of Tabellverket in 1749 provided Sweden with the oldest national population statistics in the world. The clergy, or parish ministers, were instructed to send in annual population statistics, which were first compiled at the regional level and then incorporated into the national surveys that were constructed by the Table Commission. Between 1749 and 1859, the clergy delivered parish population data, consisting of two statistical serials: In the population form, size and composition of the parish population was documented about every fifth year, and in the annual mortality form, demographical events such as births and deaths were documented. Data available from Tabellverket include for all parishes the number of men and women, number of births and number of deaths (partly also causes of death). For the 1800s, they include also socio-economic information such as household sizes, the economic standard of households, and occupational groups in the parish, although for women, the latter information is less reliable. While deaths were recorded annually, information about population size is available until 1830 for only every fifth year on average.

For the present analysis, data of the parish Uppsala Domkyrka (central Uppsala), a rather large parish at that time with several thousand inhabitants, for which complete data are available, were used.

### Climate data

We used average annual and monthly temperature and precipitation in Uppsala. Early regular recordings of temperature and precipitation were started in Uppsala around 1722, followed by Stockholm, Umeå, and Tornedalen. In a number of studies, the reliability and validity of early measurements and proxy measures have been analysed, and validated data imputed from these original measures have been provided (19, 24–27).

### Statistical methods

Average annual and seasonal temperatures were calculated based on monthly averages. No missing values occurred for monthly temperature. Cumulative precipitation per season was calculated by monthly values; if 1 month was missing, it was substituted by the mean of two other months. If 2 months were missing, the seasonal sum was missing (between 6 and 8% of the four seasons). To facilitate interpretation, we converted the sum value of precipitation from mm into cm. Winter included January and February; springtime, March to May; summer, June to August, and autumn, September to November. We excluded the climate measures of December, as they would affect annual death counts during the same year only minimally.

Year to year variability in deaths was studied in relation to annual and seasonal climate variability. We considered long-time (decennial) changes in numbers of annual deaths more likely to originate from demographic changes (population growth) and decreasing mortality rates owing to changing disease patterns and higher life expectancy. Therefore, to adjust for time-varying confounders, we de-trended annual death numbers by including the expected number of annual deaths as an offset in the regression models. The expected number of deaths was estimated by introduction of a time trend function in a regression model to explain annual death numbers. The time trends function described the mortality changes catching decadal to decadal fluctuations, and leaving year-to-year variability to be explained by the climate variability variables.

The association of annual and seasonal temperature and precipitation with the de-trended annual deaths was assessed by linear regression. For this, we applied a generalised linear model (GLM) with a negative binomial function (quasi-Poisson function), with log numbers of expected deaths as an offset variable. Models were first calculated for annual temperature and precipitation separately and together, first for both sexes, then sex-stratified. In a second step, we used seasonal temperature and precipitation separately and in combination; the full model contained climate variables for all four seasons. In a final step, the full model was calculated for three different periods separately (1749–1785, 1786–1824, and 1825–1859) to study changes in the association of climate variability and mortality over time.

Relative risks (RR) with 95% confidence intervals (CI) were calculated, α was set at 5%. *P*-values between 5 and 10% were considered borderline significant.

Based on the distribution of seasonal temperature and precipitation and the effect size of the GLM, we calculated the percentage change of death numbers for an interquartile range (IQR) increase in seasonal temperature (°C) and precipitation (cm).

The software SPSS version 19 was used to conduct the analyses.

## Results

**Fig. 1:**
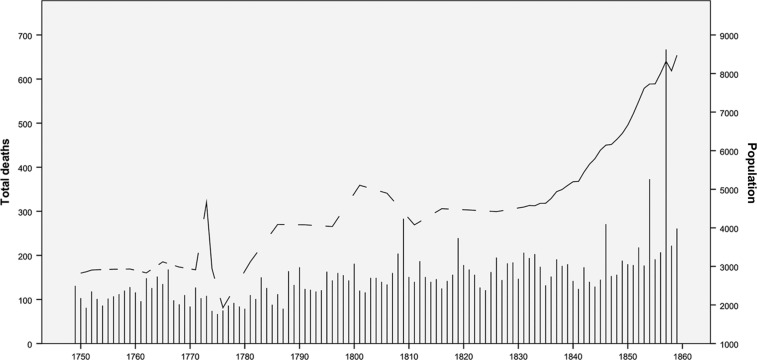
Trends in annual numbers of deaths (bars) and population size (lines), Uppsala Domkyrka parish, 1749–1859.

Between 1749 and 1859, Uppsala – like most places in Sweden – experienced high annual fluctuations in mortality, while the population increased only moderately before the 1830s, after which it almost doubled until 1859. In 1750, there were 2,826 people living in Uppsala Domkyrka parish (1,178 men and 1,648 women). Until 1859, the number had increased to 8,473 (3,857 men, 4,616 women) (Fig. 1). Unusually high numbers of deaths were observed in 1854 and 1857 (caused by cholera epidemics) and 1809 (related to the Finnish war). The number of births fluctuated between 100 and 150 per year until the 1840s, and then it almost doubled during the next two decades (data not shown).

Related to these demographic trends, the socio-economic context changed. The number of larger households (5–15 persons) increased rapidly after the 1830s; at the same time, the number of poor and very poor households decreased, and more households were rated as being well-off. More and more men were employed in the manufacturing sector, although fluctuations over time were large. In women, on the other hand, after a peak in 1800 of employment in the manufacturing sector, numbers decreased (although data on female employment are less reliable for that time). The service sector became a larger employer for both sexes since the 1840s, while there was no clear trend in the agriculture sector (data not shown).

**Fig. 2:**
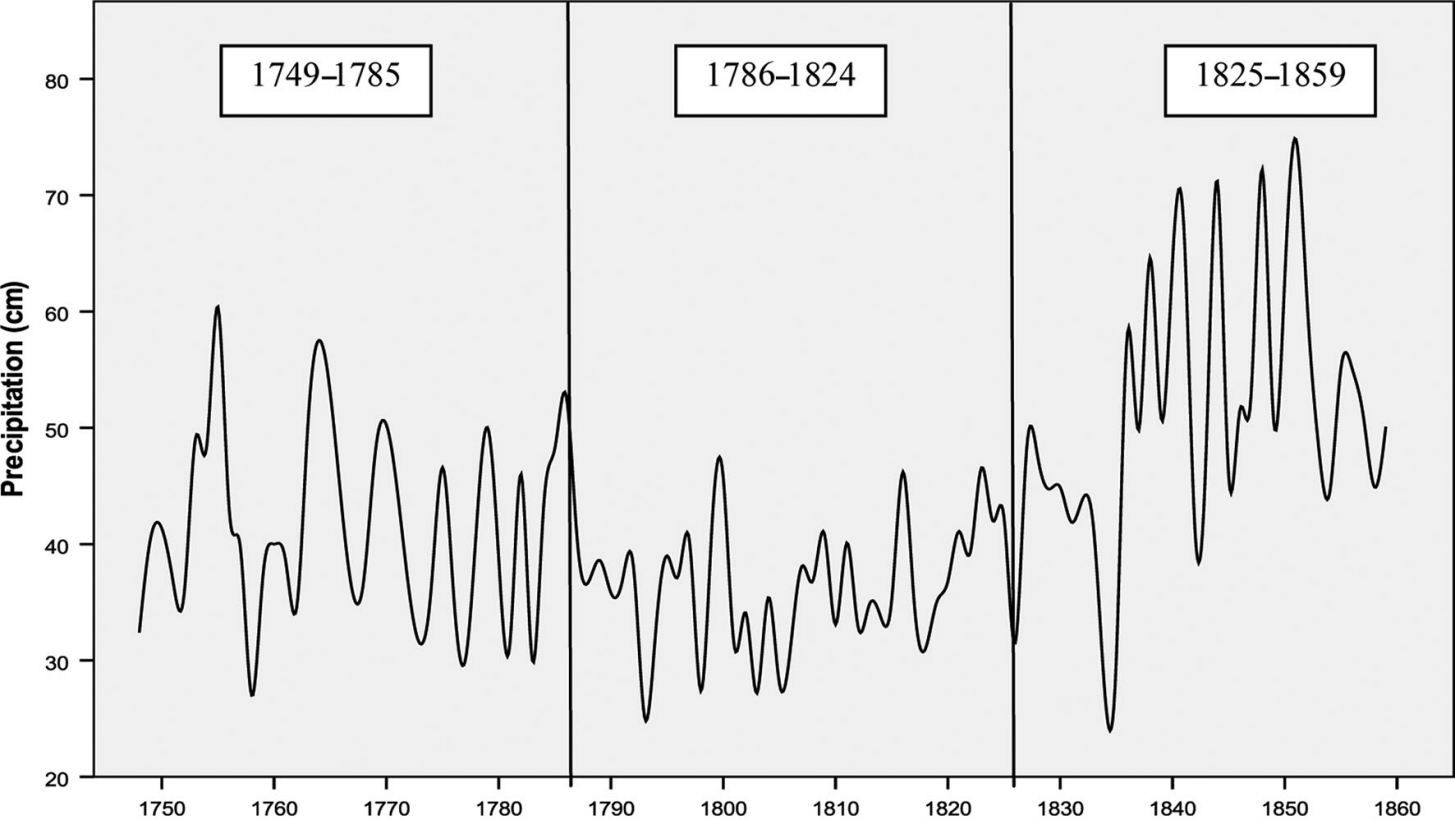
Annual precipitation (cm) in Uppsala, 1749–1859 (smooth spline).

No trends of temperatures were observed, but the climate became much wetter, especially since the 1840s (Fig. 2). Median annual precipitation was 39.9 cm in the first period (1749–1785), around 36.6 cm in the second (1786–1824), and 50.1 cm in the third period (1825–1859). Changes were largest for summer and autumn precipitation. The five coldest years were spread rather evenly over the whole period (1784, 1829, 1838, 1844, and 1856) with annual mean temperatures between 3.2 and 3.8°C, while the mean value for the whole period was 5.2°C. The five warmest years had average temperatures between 7.0 and 7.3°C. Median annual precipitation was 41 cm. The driest years were observed around 1800 with a maximum of 28 cm of precipitation per year; the five wettest years occurred between 1838 and 1851 with levels of 64.6 to 74.8 cm.

### Annual climate variability and mortality

Neither annual average temperature nor annual cumulative precipitation was statistically significantly associated with annual numbers of deaths (Table 1). When temperature and precipitation were adjusted for each other, the relative risk by temperature was 1.036/°C increase (CI 0.965–1.113, *p*=0.330) and by precipitation 1.004 per centimetre increase (CI 0.997–1.010, *p*=0.242). Also in sex-stratified analyses, no effect of annual climate was observed (Table 2).

**Table 1:** Association of annual and seasonal temperature (°C) and precipitation (cm) with annual mortality, Uppsala Domkyrka parish 1749–1859, both sexes

	Model 1 RR (95% CI)	Model 2 RR (95% CI)
Annual climate		
Annual temperature	1.036 (0.965–1.113)	–
Annual precipitation	1.004 (0.997–1.010)	–
Seasonal climate		
Winter temperature	1.007 (0.984–1.030)	1.013 (0.988–1.038)
Winter precipitation	0.992 (0.965–1.020)	0.990 (0.961–1.020)
Spring temperature	0.970 (0.935–1.007)	*0.962 (0.923–1.003)*
Spring precipitation	*0.986 (0.969–1.002)*	**0.982 (0.965–1.000)**
Summer temperature	1.025 (0.976–1.077)	*1.048 (0.997–1.102)*
Summer precipitation	1.009 (0.996–1.021)	1.007 (0.994–1.020)
Autumn temperature	1.037 (0.984–1.094)	1.031 (0.976–1.090)
Autumn precipitation	**1.013 (1.002–1.025)**	**1.018 (1.004–1.032)**

Borderline significant results (*p*=0.05–0.1) in italics. Significant results (*p*<0.05) in bold.

Model 1: Temperature and precipitation per year, respectively, per season, simultaneously included in the model.

Model 2: Full seasonal model with all seasonal climate variables simultaneously included.

RR, relative risk; CI, confidence interval.

RR values below 1.0 indicate higher temperature resp. precipitation leading to decreasing mortality, while values above 1.0 indicate increases in mortality.

**Table 2:** Association of annual and seasonal temperature (°C) and precipitation (cm) with annual mortality, Uppsala Domkyrka parish 1749–1859, sex-stratified results

	Men RR (95% CI)	Women RR (95% CI)
Annual climate*		
Annual temperature	1.030 (0.960–1.104)	1.042 (0.961–1.130)
Annual precipitation	1.003 (0.997–1.010)	1.004 (0.997–1.011)
Seasonal climate**		
Winter temperature	1.011 (0.987–1.036)	1.015 (0.986–1.045)
Winter precipitation	0.987 (0.959–1.017)	0.993 (0.959–1.027)
Spring temperature	**0.959 (0.921–1.000)**	0.965 (0.919–1.013)
Spring precipitation	*0.985 (0.968–1.002)*	**0.980 (0.960–1.000)**
Summer temperature	1.039 (0.989–1.091)	*1.058 (0.997–1.122)*
Summer precipitation	1.007 (0.994–1.019)	1.007 (0.992–1.023)
Autumn temperature	1.042 (0.986–1.100)	1.021 (0.958–1.088)
Autumn precipitation	**1.017 (1.003–1.031)**	**1.020 (1.004–1.036)**

Borderline significant results (*p*=0.05–0.1) in italics. Significant results (*p*<0.05) in bold.

* Annual temperature and precipitation simultaneously included in the model;

** Seasonal climate: All seasonal climate variables simultaneously included in the model.

RR, relative risk; CI, confidence interval.

RR values below 1.0 indicate higher temperature resp. precipitation leading to decreasing mortality, while values above 1.0 indicate increases in mortality.

### Seasonal climate variability and mortality

In the full model, all seasonal parameters were included. Precipitation during spring and autumn were statistically significantly associated with annual mortality (spring precipitation RR 0.982, CI 0.965–1.000, *p*=0.048; autumn precipitation RR 1.018, CI 1.004–1.032, *p*=0.01, per centimetre increase of precipitation). Higher springtime temperature decreased annual mortality, while higher summer temperature increased the death toll; both were however only borderline significant (*p*=0.072 and 0.068, respectively). None of the other parameters reached statistical significance. Sex-stratified analyses showed generally similar associations between the annual climate conditions and mortality for men and women, although spring precipitation reached full statistical significance only in women, while spring temperature was significantly associated with mortality only in men (Table 2).

An increase of spring precipitation by 4.4 cm (IQR) decreased the annual number of deaths by 7.6% (95% CI 0.07–14.59%), and an increase of autumn precipitation by 6.6 cm increased the number of deaths by 12.6% (95% CI 2.86–23.28%). In 1750, Uppsala Domkyrka parish recorded 103 deaths. A decrease by 7.6% would equal a reduction of that number by 8 deaths, respectively a reduction by 28/10,000 inhabitants. A 12.6% increase in annual deaths would amount to ca. 12 excess deaths in that year; or 44 excess deaths per 10,000 inhabitants.

### Climate variability and mortality in three periods

The significant effect of spring precipitation for mortality was present only in the first two periods (1749–1785 and 1786–1824), but not in the last one (Table 3). Higher winter temperature increased the annual mortality significantly only in the first period (RR 1.049, CI 1.003–1.098, *p*=0.037).

**Table 3:** Association of seasonal temperature and precipitation with annual mortality, Uppsala Domkyrka parish in three periods between 1749 and 1859, both sexes

	1749–1785 RR (95% CI)	1786–1824 RR (95% CI)	1825–1859 RR (95% CI)
Winter temperature	**1.049 (1.003–1.098)**	0.990 (0.965–1.015)	1.004 (0.942–1.071)
Winter precipitation	1.030 (0.978–1.085)	*1.045 (0.997–1.096)*	**0.941 (0.894–0.991)**
Spring temperature	0.956 (0.887–1.031)	0.978 (0.936–1.022)	0.980 (0.899–1.069)
Spring precipitation	**0.972 (0.947–0.999)**	**0.965 (0.935–0.995)**	0.999 (0.967–1.031)
Summer temperature	1.027 (0.946–1.114)	1.017 (0.960–1.078)	*1.107 (0.993–1.235)*
Summer precipitation	0.995 (0.977–1.014)	0.996 (0.976–1.016)	0.994 (0.967–1.021)
Autumn temperature	0.970 (0.886–1.062)	1.019 (0.948–1.094)	1.044 (0.934–1.166)
Autumn precipitation	0.997 (0.962–1.034)	1.000 (0.980–1.021)	*1.024 (0.997–1.052)*

Borderline significant results (*p*=0.05–0.1) in italics. Significant results (*p*<0.05) in bold.

All seasonal climate variables simultaneously included in the model.

RR, relative risk; CI, confidence interval.

RR values below 1.0 indicate higher temperature resp. precipitation leading to decreasing mortality, while values above 1.0 indicate increases in mortality.

Neither in the first nor the second period, autumn precipitation showed significant associations with mortality. However, the significant effect of autumn precipitation, which was found for the whole observation time, was mainly due to its relevance during the last period, 1825–1859 (RR 1.024 per cm, CI 0.997–1.052, *p*=0.08). Thus, alhough this association was only borderline significant, it was comparably large. At the same time, winter precipitation was found to decrease mortality statistically significantly (RR 0.941 per cm, CI 0.894–0.991, *p*=0.02).

## Discussion

In this study, we used aggregated data to investigate the relationship between climate variability and annual death counts in Uppsala Domkyrka parish between 1749 and 1859. We found statistically significant associations of springtime and autumn precipitation with mortality. While rainfall in spring decreased the number of deaths, precipitation during autumn increased the number. High levels of rainfall in spring is most likely beneficial for the growth of crops, while during autumn it might lead to rotting of the harvest, causing food shortage and food-borne diseases. This might explain the opposite effects of spring and autumn rain; however, it is unclear why the association of autumn rain with mortality was not observed during the first two periods (i.e. 1749–1824). In the last period, when the association was largest, overcrowding in urban settlements had become a more common phenomenon, giving probably rise to the spread of airborne diseases such as influenza. At that time, a humid autumn climate could have had an impact on mortality through such diseases rather than because of harvest failure with following malnutrition and food-borne diseases. Analyses of cause-specific mortality on an annual or monthly basis could bring more light into this issue; available data for this parish are, however, limited.

The protective effect of spring precipitation during the 18th and early 19th century was not present during the last decades (1825–1859). In the mid-1800s, average precipitation was much higher than in the decades before; therefore, the association with mortality might be absent because rainfall reached sufficient levels in all or most years of that period. Another explanation can be the decreasing dependency of the population on local agricultural production or a change to crops that were less sensitive to spring time conditions. However, Sundin and Willner observed that a strong connection between harvest and mortality, partly via local food prices, was present up to the 1860s in Sweden (2). Although then more food in general was available than in the centuries before, it was unevenly distributed between the rich and the poor, and the poor were likely most affected.

Temperature variability appeared to be a less relevant factor for mortality than precipitation during the 18th and 19th century. In our fully adjusted model, the effect of spring and summer temperature on mortality reached borderline significance, although in opposing directions. While in spring a warmer climate reduced the mortality, it led to more deaths in summer. Again, agricultural productivity can be a partial explanation. Diverse records from Europe show the relationship between low average temperature, harvest failures, food prices, famines, and epidemics (18, 20). However, also practice of food storage hygiene in combination with more efficient reproduction of pathogens may explain this. During periods of excessive heat (heat waves), mortality has been observed to increase generally in almost all causes in recent time (28).

In Sweden, a warm spring with good rainfall will increase the chance of a rich harvest, on which the pre-industrial urban population was dependent. Relatively hot summers, on the contrary, might facilitate the spread of infectious agents and their vectors, leading to higher infectious disease mortality (29, 30).

Environmental and socio-economic factors for diseases are mutually interdependent. Weather, for example, influences mortality through social pathways – unfavourable weather conditions might lead to crop failure and poverty, which in turn cause malnutrition and high mortality rates especially in young children. Galloway (31) describes changes in vital rates in line with changes in solar activity and annual temperature in England between 1250 and 1800. During the 17th century, England, like other European countries, experienced a drastic decline in average temperature, which was accompanied by shorter life expectancies, outmigration, and falling gross reproduction rates. The huge outbreak of the Tambora volcano in Indonesia in 1815, followed by several years of global cooling by 2–3°C, led to harvest failures in Europe, China, and the Americas. In some European countries, food riots and social unrest occurred as a consequence of increased food prices, starvation, and outbreaks of typhus; and large migration contributed to the spread of infectious diseases to other regions and countries (20). On the other hand, social change – improved hygienic living conditions and industrialisation – changed human relationships with nature and reduced the dependency on favourable climate conditions, and through this mechanism had a tremendous impact on life expectancy and reduced mortality rates. The climate impact on health took place in the context of rapidly changing social conditions, which has to be taken into account when interpreting associations between climate variability and mortality.

Our analyses were conducted with population data of Uppsala Domkyrka parish, an urban setting in central Sweden. At that time, living conditions and mortality rates were much higher in towns than in rural areas. Hence, the association of mortality with climatic factors is most likely to be different in the countryside, and possibly also modified by geographical region.

### Strength and limitations

The digitised Tabellverket population data and the early records of temperature and precipitation in Uppsala before the start of regular measurements gave us the unique opportunity to investigate the relationship between climate and mortality over more than 100 years prior to industrialisation in Sweden. This enabled us to look at long-term effects of climate variability on annual mortality, rather than weather variability on a daily or weekly scale.

However, the available data and the presented analyses have some limitations. When investigating the association of seasonal temperature and precipitation with annual deaths, the true effect of climate, especially for autumn climate, might be underestimated, since many of the observed deaths occurred prior to exposure. Autumn climate that leads to failing harvests possibly does not show its full impact on mortality before the next winter or spring, when food reserves are running out. In seasonal models, December temperature and precipitation measures were omitted since we assumed that they will not have a substantial effect on the current year's mortality. However, their association with the next year's death numbers is not accounted for in our models. Further analyses therefore shall investigate monthly rather than annual deaths as the main outcome, quantify different lag times between exposure and events, and consider cross-annual effects.

There might be a non-linear relationship between climatic factors and mortality, which was not assessed in our analyses. This might lead to an underestimation of true effects. It is possible that temperature or precipitation have opposing effects on different causes of death. A cold winter, for example, is known to increase cardiovascular and respiratory mortality (14, 17), while simultaneously it might reduce the incidence of vector-borne diseases in the months to follow.

The analyses build on parish registries recorded centuries ago by church ministers. Although the validity of these entries is unknown, it can be assumed that errors regarding annual deaths and population numbers were more or less random. Hence, the risk of bias can be considered small, and observed associations between climate parameters and annual numbers of deaths are likely to underestimate the true effects.

We did not control for other time varying factors beyond the adjustment for decadal time trends. If such factors with substantial year-to-year variability are associated with climate variability and mortality, they might confound the observed results.

## Conclusions

In urban Uppsala during the 18th and 19th century, precipitation appeared to be a stronger predictor for mortality than temperature. Higher spring precipitation decreased and autumn precipitation increased the number of deaths, but this association was different before and during the early stages of industrialisation. Sufficient rainfall during the spring would most likely increase agricultural production, a factor which became less decisive for survival in the process of industrialisation. This might explain the lack of association between spring precipitation and mortality in the last period (1825–1859). On the contrary, higher amounts of autumn rain (which overall was at high levels during the last decades of the study period) might have facilitated disease transmission related to hygiene practise and indoor crowding. Rainfall might also lead to rotting of harvest and thereby contribute to higher mortality rates. Different pathways from climate variability to mortality have to be considered, accounting for different societal and socio-economic circumstances, and during different seasons.

These historical records of population and meteorological data can highlight the role of environmental factors on human health at the time of demographic and epidemiological transition. More sophisticated models should take changes in socio-economic conditions and agricultural practices during that time into account to analyse opposing or synergetic effects on mortality. Further analyses should also investigate age-specific climate impacts, as well as cause-specific mortality to understand better causal mechanisms in the relation between climate variability and health.
